# Response: Commentary: The Scavenger Receptor SSc5D Physically Interacts with Bacteria through the SRCR-Containing N-Terminal Domain

**DOI:** 10.3389/fimmu.2017.01004

**Published:** 2017-08-23

**Authors:** Liliana Oliveira, Alexandre M. Carmo

**Affiliations:** ^1^i3S – Instituto de Investigação e Inovação em Saúde, Universidade do Porto, Porto, Portugal; ^2^IBMC – Instituto de Biologia Molecular e Celular, Porto, Portugal

**Keywords:** scavenger receptor cisteine-rich, CD6, bacteria, pattern recognition receptors, surface plasmon resonance

While until recently there were no known common functional features shared between different scavenger receptor cysteine-rich (SRCR) group B glycoproteins, between 2000 and 2009 the receptors DMBT1, Spα, CD6, and CD163 were reported to bind bacteria, thus suggesting a potential broad role of SRCR proteins as pattern recognition receptors (PRRs) (1–4). The reports came from three different teams, each using different approaches and in the context of their specific research.

Having cloned the five-SRCR domain-containing soluble protein SSc5D (5), we addressed in the paper by Bessa Pereira et al. (6) a possible PRR function for SSc5D, but introduced additionally a question not commonly asked: do all SRCR proteins bind equally to the same bacteria strains or species? The very fact that the different authors published their reports logically means that their studied receptors did bind bacteria; but how each receptor fared comparing with the others in the bacteria-binding properties was not fully weighed.

To perform an unbiased analysis of binding to bacteria of the SRCR domain-containing parts of CD5, CD6, Spα, and SSc5D, we produced all receptors using the same vectors introducing the same tags and used the same mammalian expression and protein purification systems. The analysis was performed using two methods, in the first incubating proteins with bacteria, followed by lysis and protein detection using immunoblotting, and in the second using the more sensitive technique surface plasmon resonance (SPR).

Given the impartial and balanced nature of our study, we were surprised to understand that Lozano and Martínez-Florensa consider in their commentary that we “cast doubt on the well-documented bacterial-binding properties of CD6.” This statement is simply not accurate, because analyzing the spirit of our paper and reinforced in its conclusions, we never challenged Lozano’s previous findings. On the contrary, we have always assumed as definitive that CD6 can recognize and bind to bacteria in several of our publications, including a recent editorial (7).

The fact that in one of the methods we used, the traditional protein–bacteria binding assays, we did not detect interactions between recombinant CD6 with the *E. coli* and *L. monocytogenes* strains tested does not change our perception of the bacteria-binding potential of CD6. Relevantly, this observation was produced at the very same stage in the paper where we were also not able to detect interactions of our own query receptor SSc5D with *Listeria* and one of the *E. coli* strains. A main aim of the study was precisely to develop a more sensitive and reliable novel method to tackle a difficult and controversial problem. The conclusions of the SPR analysis and thus of our paper are categorical in that the binding of CD6 to the tested *E. coli* RS218 and *L. monocytogenes* EGD-e strains is “clearly above the level of the sCD5 negative profile.”

Lozano and Martínez-Florensa’s commentary contains other factual inaccuracies. We find bizarre the argument that our alleged doubts are “Exclusively based on a single experimental evidence” when in fact our study involved two types of experiments. Also, the suggestion that we used tetrameric instead of unconjugated CD6 for bacteria-binding assays is incorrect: all recombinant SRCR receptors, including CD6, were used as monomeric proteins in the receptor–bacteria binding assays and nothing in the text from start to finish could suggest otherwise or mislead the reader. Tetrameric CD6 was assembled as a cytometry useful reagent in supplementary data for the sole purpose of confirming that recombinant CD6 retained its natural ability to bind the membrane-expressed ligand CD166, as we had previously demonstrated (8).

Why, then, are our and Lozano’s results not concordant in one type of experiment? There are countless possible reasons given that the experimental models differ in a number of aspects, such as that we detected the bacteria-bound proteins using anti-HA primary antibodies followed by secondary HRP-conjugated goat anti-mouse antibodies, whereas they biotinylated their proteins and detected them using HRP-conjugated streptavidin. However, this does not explain why by comparison we could easily detect Spα and SSc5D, but not CD6, binding to bacteria. Although unlikely, it is also possible as they suggest that the introduction of tags could impede any CD6 binding to bacteria; but the exact same modifications were introduced in Spα and SSc5D as well. A simpler straightforward possibility to explain the different patterns of binding is that the bacteria strains used are not the same.

As it is shown in our paper and also illustrated in previous studies, the binding profiles of a given receptor to different strains of a same bacterial species can vary dramatically (1, 6). Therefore and objectively, using receptors produced by the same way and incubated with the same bacteria in identical conditions, we can state that in our system Spα and N-SSc5D attach better to the bacterial strains used than does sCD6. This is consistent with the notion that different SRCR may have dissimilar pathogen recognition spectra, or that some are more specialized in bacteria recognition than others. None of this excludes that CD6 interacts with bacteria.

However, it should be noted that for CD6 to have a biological protective function, there needs to be no proportional correlation of the binding strength to pathogens namely when the model addressing the protective effect of CD6 is sepsis. As Lozano and colleagues convincingly described, CD6 protects mice from LPS-induced septic shock and from polymicrobial sepsis (3, 9). While this effect can be mediated by direct binding of sCD6 to LPS and/or bacteria, which by aggregation could facilitate clearance and consequently lead to lower inflammatory cytokine release, a strong anti-inflammatory role of CD6 *per se* ought not to be excluded. By competing with T cell-surface CD6 binding to antigen-presenting cell (APC)-expressed CD166, sCD6 may hamper or weaken T–APC interactions, thus diminishing inflammatory responses and having an impact on the outcome of the septic process.

Notwithstanding the widely demonstrated pathogen-sensing properties of CD6, or likewise of Spα or SSc5D, to reduce their prophylactic or curative function to the microbe-binding properties is, in our opinion, an oversimplification. The biological functions of soluble circulating SRCR proteins will undoubtedly be further clarified in the near future.

## Author Contributions

LO and AC wrote the response to the commentary.

## Conflict of Interest Statement

The authors declare that the research was conducted in the absence of any commercial or financial relationships that could be construed as a potential conflict of interest.

## References

[1] Prakobphol A Xu F Hoang VM Larsson T Bergstrom J Johansson I Salivary agglutinin, which binds *Streptococcus mutans* and *Helicobacter pylori*, is the lung scavenger receptor cysteine-rich protein gp-340. J Biol Chem (2000) 275(51):39860–6. 10.1074/jbc.M006928200 11007786

[2] Sarrias MR Roselló S Sánchez-Barbero F Sierra JM Vila J Yélamos J A role for human Sp alpha as a pattern recognition receptor. J Biol Chem (2005) 280(42):35391–8. 10.1074/jbc.M505042200 16030018

[3] Sarrias MR Farnós M Mota R Sánchez-Barbero F Ibáñez A Gimferrer I CD6 binds to pathogen-associated molecular patterns and protects from LPS-induced septic shock. Proc Natl Acad Sci U S A (2007) 104(28):11724–9. 10.1073/pnas.0702815104 17601777 PMC1913855

[4] Fabriek BO van Bruggen R Deng DM Ligtenberg AJ Nazmi K Schornagel K The macrophage scavenger receptor CD163 functions as an innate immune sensor for bacteria. Blood (2009) 113(4):887–92. 10.1182/blood-2008-07-167064 18849484

[5] Gonçalves CM Castro MA Henriques T Oliveira MI Pinheiro HC Oliveira C Molecular cloning and analysis of SSc5D, a new member of the scavenger receptor cysteine-rich superfamily. Mol Immunol (2009) 46(13):2585–96. 10.1016/j.molimm.2009.05.006 19535143

[6] Bessa Pereira C Bockova M Santos RF Santos AM de Araujo MM Oliveira L The scavenger receptor SSc5D physically interacts with bacteria through the SRCR-containing N-terminal domain. Front Immunol (2016) 7:9 10.3389/fimmu.2016.00416 27790215 PMC5061727

[7] Carmo AM Heads or tails: betting on CD6 as a resurged target for autoimmune diseases and sepsis. Curr Drug Targets (2016) 17(6):618 10.2174/138945011706160324153314 27017998

[8] Oliveira MI Gonçalves CM Pinto M Fabre S Santos AM Lee SF CD6 attenuates early and late signaling events, setting thresholds for T-cell activation. Eur J Immunol (2012) 42(1):195–205. 10.1002/eji.201040528 21956609 PMC3298641

[9] Martínez-Florensa M Consuegra-Fernández M Aranda F Armiger-Borràs N Di Scala M Carrasco E Protective effects of human and mouse soluble scavenger-like CD6 lymphocyte receptor in a lethal model of polymicrobial sepsis. Antimicrob Agents Chemother (2017) 61(1):e01391–16 10.1128/AAC.01391-16 27895015 PMC5192129

